# Cloning, expression and functional activity of deoxyhypusine synthase from *Plasmodium vivax*

**DOI:** 10.1186/1471-2180-6-91

**Published:** 2006-10-16

**Authors:** James T Njuguna, Marwa Nassar, Achim Hoerauf, Annette E Kaiser

**Affiliations:** 1 Institute for Medical Microbiology, Immunology and Parasitology, D-53105 Bonn, Germany; 2 International Livestock Research Institute (ILRI), Naivasha road, P.O. Box 30709, Nairobi, Kenya; 3 Theodor Bilharz Research Institute, Warak El-Hadar, Kornish El Nile P.O. Box 30 Imbaba, Giza 12411, Egypt; 4 The German University in Cairo, New Cairo City, Main Entrance of Al Tagamoa Al Khames, Egypt

## Abstract

**Background:**

*Plasmodium vivax *is the most widespread human malaria parasite. However, genetic information about its pathogenesis is limited at present, due to the lack of a reproducible *in vitro *cultivation method. Sequencing of the *Plasmodium vivax *genome suggested the presence of a homolog of deoxyhypusine synthase (DHS) from *P. falciparum*, the key regulatory enzyme in the first committed step of hypusine biosynthesis. DHS is involved in cell proliferation, and thus a valuable drug target for the human malaria parasite *P. falciparum*. A comparison of the enzymatic properties of the DHS enzymes between the benign and severe *Plasmodium *species should contribute to our understanding of the differences in pathogenicity and phylogeny of both malaria parasites.

**Results:**

We describe the cloning of a 1368 bp putative deoxyhypusine synthase gene (*dhs*) sequence from genomic DNA of *P. vivax *PEST strain Salvador I (Accession number AJ549098) after touchdown PCR. The corresponding protein was expressed and functionally characterized as deoxyhypusine synthase by determination of its specific activity and cross-reactivity to human DHS on a Western blot.

The putative DHS protein from *P. vivax *displays a FASTA score of 75 relative to DHS from rodent malaria parasite, *P. yoelii*, and 74 relative to that from the human parasite, *P. falciparum *strain 3D7. The ORF encoding 456 amino acids was expressed under control of IPTG-inducible T7 promoter, and expressed as a protein of approximately 50 kDa (theoretically 52.7 kDa) in *E. coli *BL21 DE3 cells. The N-terminal histidine-tagged protein was purified by Nickel-chelate affinity chromatography under denaturing conditions. DHS with a theoretical pI of 6.0 was present in both eluate fractions. The specific enzymatic activity of DHS was determined as 1268 U/mg protein. The inhibitor, N-guanyl-1, 7-diaminoheptane (GC7), suppressed specific activity by 36-fold. Western blot analysis performed with a polyclonal anti-human DHS antibody revealed cross-reactivity to DHS from *P. vivax*, despite an amino acid identity of 44% between the proteins.

**Conclusion:**

We identify a novel DHS protein in the more benign malaria parasite,*P. vivax*, on the basis of specific enzymatic activity, cross-reactivity with a polyclonal antibody against human DHS, and amino acid identity with DHS homologs from the rodent malaria parasite, *P. yoelii*, and human *P. falciparum *strains.

## Background

*Plasmodium vivax *is the most widely distributed human malaria parasite and infects 70–80 million individuals per year worldwide. In most malaria endemic regions, *P. falciparum and P. vivax *infections coexist, and mixed infections are prevalent. Interactions between the two parasites determine the outcome of the disease and future therapeutic perspectives with regard to infection control. However, there is limited knowledge about the biology of the *P. vivax *parasite at present, due to the lack of an *in vitro *culture system.

To accelerate drug discovery for the more benign *P. vivax*, genome sequencing of the malaria parasite was performed to facilitate its annotation [1]. A transcriptome approach [2] was applied to study gene expression in the mixed erythrocytic stages of a *P. vivax *field isolate. Transcribed genes (30%) displayed significant homology to those involved in metabolism and ribosomal proteins consistent with the trophozoite stage of the field strain.

Over recent years, increasing resistance of malaria parasites to therapy, and in particular, re-emergence of *P. vivax *infections, has increased the need to develop strategies of identifying novel drug targets. The polyamine pathway has been exploited for antiparasitic chemotherapy. Our research is focused on the formation of the novel amino acid hypusine, which involves two steps. In the first step, deoxyhypusine synthase (DHS) [EC 1.1.1.249] transfers an aminobutyl moiety from the triamine spermidine to a specific lysine residue in the eIF-5A (eukaryotic initiation factor 5A) precursor protein to form deoxyhypusine. In the second step, deoxyhypusine hydroxylase (DHH) [EC 1.14.9929] completes hypusine biosynthesis through hydroxylation. Deoxyhypusine modification is important for the viability of eukaryotic cells [3]. We have recently cloned two genes involved in hypusine biosynthesis from the malaria parasites *P. falciparum *and *P. vivax*, specifically, deoxyhypusine synthase (DHS) and eukaryotic initiation factor 5A (EIF-5A). To elucidate the phylogenetic relationship between deoxyhypusine synthase (DHS) proteins from both parasitic species, we clone the putative *dhs *gene from *P. vivax*, and determine its specific enzymatic activity.

## Results

### Nucleotide sequence of a putative dhs gene from *P. vivax*

The DHS amino acid sequence of *P. falciparum *strain NF 54 [4] was used to screen a library of expressed sequence (EST) tags from *P. vivax *strain PEST Salvador I. Based on the 5' and 3' ends of *dhs*, two primers were constructed to amplify the 1368 bp sequence encoding 456 amino acids from the genomic DNA of *Plasmodium vivax *strain PEST Salvador I. The purified fragment was subcloned into pSTBlue-1**A**ccep**T**or vector, and sequenced (accession number AJ549098). The *dhs *gene has an AT content of 52%, and a comparable GC content of 48%.

**Figure 1:**
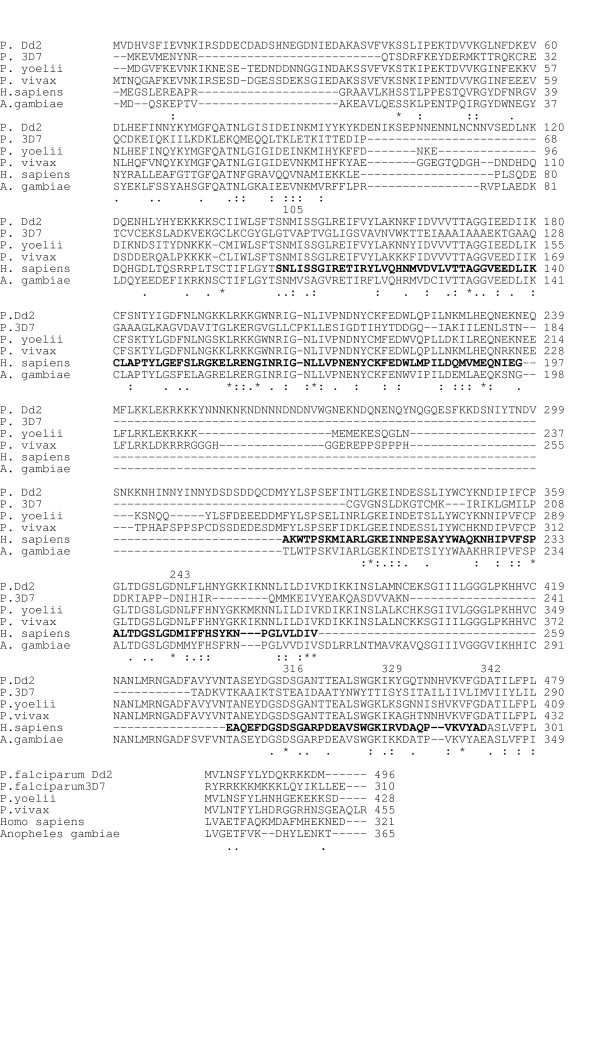
**Amino acid alignment between a putative DHS protein from *P. vivax *and two different P. falciparum strains, the rodent malaria parasite *P. yoelii*, human and the mosquito *Anopheles gambiae***. Numbering refers to DHS in the two P. falciparum species. Lane 1: P. falciparum strain Dd2 (accession number AF290977); lane 2: P. falciparum strain 3D7 (accession number NC_004317); lane 3: *P. yoelii *(XM-724232); lane 4: *P. vivax *(AJ549098) ; lane 5: human (U26266), lane 6: *Anopheles gambiae *(XM-316567). Gaps (-) were introduced to obtain maximum alignment. Arterisks label amino acid identities, colons (:) and dots (.) label amino acid similarities. The spermidine binding site (243–329 refering to human DHS numbering) is marked by bold amino acids. The NAD binding site from serine105 to aspartic acid 342 is marked in bold letters. The active center of the DHS protein from glutamine 324 to lysine 329 is bolded black.

The deduced amino acid sequence of the *P. vivax *protein was aligned with different putative DHS sequences from other *Plasmodium *species, i. e., *P. falciparum *Dd2 and 3D7 strains (Fig. 1, lanes 1 and 2), rodent malaria parasite *P. yoelii *(Fig. 1, lane 3), and *Anopheles gambiae *(Fig 1, lane 6). The *P. vivax *DHS amino acid sequence displayed FASTA scores of 74 and 73 relative to the *P. falciparum *strains Dd2 and 3D7, respectively. Notably, *P. vivax *DHS exhibits 66% amino acid identity between positions 1–236 and 91% identity between positions 276–443 with the *P. falciparum *Dd2 strain.

There are several gaps in the amino acid stretches at positions 103, 240, 257 and 442 in the *P. vivax *DHS protein, which are absent in the *P. falciparum *homolog (numbering refers to the amino acid sequence from *P. vivax*). The *P. vivax *DHS protein has a FASTA score of 75 to its homolog from the rodent malaria parasite *P. yoelii*, while amino acid identity to the human DHS protein (Fig. 1, lane5) is only 44%. The lowest FASTA score of 49 was obtained between *P. vivax *and *Anopheles gambiae *DHS proteins (Fig. 1, lane 6)

### Expression of *P. vivax *DHS protein in *E. coli *cells and affinity purification

**Figure 2:**
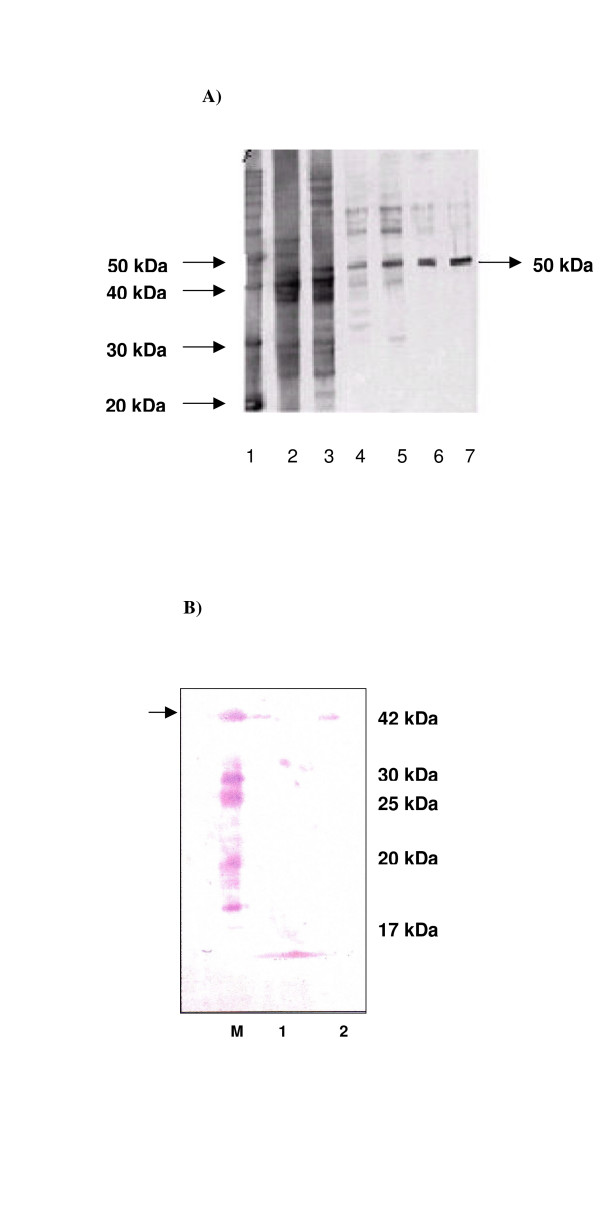
**A: Expression and purification of recombinant putative six histidine tagged deoxyhypusine synthase from *P. vivax *by Nickel-chelate chromatography**. 12% SDS PAGE protein gel: 1) protein marker: 10 kDa ladder; 2) non induced bacterial cell lysate 3) induced bacterial cell lysate with 100 mM IPTG after 4 hours of induction; 4 and 5) wash fractions obtained after purification; 5 and 6) eluate fractions of purified putative deoxyhypusine synthase. B: Western Blot analysis of the expressed, purified *P. vivax *DHS protein. lane 0) standard molecular size marker (Roth); lane 1) protein extract of purified DHS protein from *P. vivax *by Nickel-chelate chromatography. lane 2) protein extract of purified human DHS protein. The blot was probed with a 1: 800 diluted polyclonal antiserum against human DHS.

The purified *P. vivax *DHS protein has a calculated molecular size of 50 kDa (theoretically 52.7 kDa), and a pI value of 6.0. Initially, primers#1 and #2 containing *Nde*I and *Bam*HI restriction sites, respectively, were used for amplification of *dhs*, which was cloned into the pET15b vector for protein expression. Protein extracts prepared after 4 h of induction with IPTG exhibited a prominent band of approximately 50 kDa on an SDS-PAGE gel (Fig. 2A, lane 3). In contrast, no band was detectable in the uninduced control culture (Fig. 2A, lane 2.) The recombinant protein was expressed with a six-histidine tag at the amino-terminus, allowing purification by Nickel-chelate affinity chromatography. Purified protein was analyzed by SDS-PAGE (Fig. 2). The protein was detected at 50 kDa in both eluate fractions (Fig. 2, lanes 6 and 7).

### Modification of *P. vivax *eIF-5A precursor protein by *P. vivax *deoxyhypusine synthase

For the deoxyhypusine synthase assay, the purified eIF-5A precursor protein and DHS enzyme from *P. vivax *were applied [5]. The incorporation of radioactively labeled spermidine into the substrate precursor protein was assayed using a filter paper assay [14]. Purified samples were immediately used to determine specific enzymatic DHS activity. The average specific enzymatic activity from two experiments was 1268 U/mg protein suggesting that DHS protein from *P. vivax *is able to modify the eIF-5A precursor protein (Table 1, column A). In a control experiment, no specific enzymatic activity was detected when water was used instead of DHS (Table 1, column B). The specific enzymatic activity of *P. vivax *DHS was suppressed 36-fold with the inhibitor N-guanyl-1,7-diaminoheptane (GC7) (Table 1, column C).

### Plasmodium vivax deoxyhypusine synthase shows cross-reactivity to its human homolog

To determine whether a polyclonal antibody against human DHS recognized the corresponding protein from *P. vivax*, the Nickel-chelate purified protein was subjected to Western blot analysis (Fig. 2B, lane 1). As a positive control, we used purified human DHS protein (Fig. 2B, lane 2). The antibody specifically recognized human DHS protein with a molecular size of 41 kDa (Fig. 4, lane 2), and cross-reacted with the *Plasmodium vivax *DHS protein (Fig. 2B, lane 1).

## Discussion

Reduced *in vitro *susceptibility [6] of field isolates from *Plasmodium falciparum *strains to artemeter derivatives is an alarm signal necessitating the rapid deployment of drug combinations. There is an urgent need to elucidate the genome of human malaria parasites. In 2000, after the completion of the *P. falciparum *genome project, the sequencing of expressed sequence tags (EST) from the human malaria parasite *P. vivax *of Salvador PEST strain I, isolated from a malaria patient from Thailand, was initiated. No information about this parasite is available to date. In contrast to *P. falciparum*, it infects reticulocytes, which are young immature erythrocytes. Due to the lack of a reproducible continuous *in vitro *culture system [7], detailed molecular analyses were not possible.

Recently, we exploited the biosynthetic pathway of the generation of the novel amino acid, hypusine, in the human malaria parasite *P. falciparum *[8]. Two proteins, i.e., DHS and eukaryotic initiation factor 5A (eIF-5A) involved in the formation of deoxyhypusine, the first committed step in the hypusine biosynthetic pathway, are potential new drug targets in *Plasmodium falciparum *[9]. To identify the phylogenetic homologs of the *dhs *gene in different *Plasmodium *species, a library of *Plasmodium vivax *expressed sequence tags was screened for a DHS homolog from *P. falciparum*. The putative DHS sequence from *P. vivax *is encoded by a 1386 bp nucleic acid sequence, with the most significant FASTA scores of 75 to *P. yoelii *and 74 to *P. falciparum *strain 3D7. These results are in agreement with initial comparative analyses of the *P. vivax *genome with the other related *Plasmodium *species, which revealed significant conservation of gene synteny between the different species within chromosome cores, in contrast to subtelomeric regions displaying reduced homology [10].

*P. vivax *DHS displays striking amino acid sequence identity to its homolog from *P. falciparum*. Notably, 89% amino acid identity is observed between *P. vivax *DHS protein (Fig. 1, lane 4) and the human homolog between residues 290–308 (human DHS numbering) (Fig. 1, lane 5). However, several gaps appear in the *P. vivax *protein sequence at positions 103, 240, 257 and 442, which are absent in the *Plasmodium falciparum *homolog.

The most significant differences between human and *Plasmodium *DHS homologs are evident in the spermidine binding site (residues 243–342, human DHS numbering), although the active site is highly conserved. The active site includes a conserved region of six amino acids from Glu 323 to Lys 329 (human DHS numbering) (Fig. 1) [11,12]. Asp 316, one of the charged amino acids at the bottom of the active site tunnel responsible for binding to spermidine and the GC_7 _inhibitor, is highly conserved in DHS proteins in *Plasmodium *[13]. Moreover, in both DHS proteins, Gly 314 is within hydrogen-binding distance of the guanidinium group [13].

The NAD-binding site (positions 105–342, human DHS numbering) is strongly conserved between the different *Plasmodium *species and their human homologs. However, a stretch of 85 amino acids mainly comprising asparagine in this site is missing in DHS homologs from *Plasmodium*.

To confirm the function of *P. vivax *DHS, we determined its specific enzymatic activity in modification of eukaryotic translation initiation factor, eIF-5A, by incorporation of the aminobutyl-moiety of ^14^[-C]-spermidine as a substrate (Table 1, column A). Specific enzymatic activity was determined as 1268 U/mg protein, which was 9-fold less than that calculated by Molitor and colleagues (2004) [14], who used human DHS protein for the modification of eIF-5A from *P. falciparum*. In a control experiment with water instead of DHS from *P. vivax*, no specific enzymatic activity was detected.

In a parallel inhibition experiment, the most commonly used competitive inhibitor of DHS, N1-guanyl-1,7-diaminoheptane (GC7) (Table 1, column C), was employed. GC7 inhibits DHS in a competitive manner by binding to its active site [15], as observed in crystallized form II that was recently obtained for the complex of DHS and GC7. This inhibitor reduced specific enzymatic activity by 36-fold (Table 1, column C).

While the amino acid identity between human and *Plasmodium vivax *DHS is only 44%, we detected cross-reactivity between a polyclonal antibody raised against the human DHS enzyme and the expressed Nickel chelate-purified protein (Fig. 2B, lane 1) from *P. vivax*, suggesting that the anti-DHS antibody recognizes specific epitopes present in both proteins.

## Conclusion

The putative *P. vivax *DHS protein consists of 456 amino acids with significant FASTA scores of 75 to *P. yoelii *and 74 to *P. falciparum *strain 3D7 DHS proteins. A specific DHS enzymatic activity assay with ^14^C-labeled spermidine as the substrate and inhibition by N-guanyl-1,7-diaminoheptane (GC7) confirmed functional activity. Moreover, the DHS protein from *P. vivax *cross-reacted with a polyclonal antibody against anti-human DHS. Further biochemical characterization analyses of the *P. vivax *DHS protein are currently underway.

## Methods

### Amplification of dhs from genomic DNA of *P. vivax *strain PEST Salvador I

The amplification reaction comprised 89 ng genomic DNA of *P. vivax *strain PEST Salvador I, 200 pmol forward primer 1# ATG ACG AAC CAA GGG GCT TTT and reverse primer 2# TCA CCT GAG CTG CGC TTC ACC, 10 mM dNTP, 75 mM MgCl_2_, 2 μl 10 fold PCR buffer and 5 U *Taq *polymerase (Qiagen) in a total volume of 20 μl. Amplification was performed using a temperature profile of 94°C for 5', 94°C for 1', 60°C for 1', 72°C for 1' for 30 cycles, and 72°C for 10', generating a product of 1386 bp. The PCR product was subcloned into the pSTBlue-1 **A**ccep**T**or Vector (Novagen). Positive recombinant clones were identified by dideoxynucleotide sequencing (MWG Biotech, Munich).

### Expression and purification of DHS protein from *P. vivax *by Nickel-chelate affinity chromatography

After purification (Quiaex), the 1386 bp fragment was subcloned into petBlue-1 vector (Novagene), and transformed for expression in Tuner™ (DE3) pLacI cells (Novagen). Insertion of the open reading frame in the correct orientation was confirmed by sequencing (MWG Biotech, Munich). Cells with an optical density of 0.6–1.0 at OD_600 _were resuspended in 3 ml of fresh Luria Bertani (LB medium) supplemented with 0.5–1.0 % glucose and 100 μg/ml ampicillin were used to inoculate 100 ml LB-medium with the same concentration of supplements at 37°C until OD_600 _0.5–1.0. Prior to induction, cells were split into two 50 ml cultures. One culture was supplemented with 500 μl of 100 mM IPTG, while the other served as the uninduced control. Protein extracts were prepared 4 h after induction by centrifugation, lysed, heated at 100°C, and run on a 12% SDS polyacrylamide gel at 100 V. The 10 kDa protein ladder (Gibco BRL) and RainBow Marker were used as molecular weight markers.

*P. vivax *DHS protein was expressed under control of the T7-inducible promoter in pET15b vector (Novagene), which allows purification of 6xHis-tagged proteins. PST-Blue 1 **A**ccep**T**or**V**ector (Novagene) containing the *dhs *gene was used as a template for subcloning the full-length *dhs *sequence by amplification with expression primers (forward 1# 5'-AAC CCC ATA TGA CGA ACC AAG GGG CTT TT-3' containing an *Nde *I restriction site (underlined) and a *Bam *HI restriction site (underlined; reverse 2 # 5'-AAAA GGA TCC TCA CCT GAG CTG CGC TTC-3'), respectively. Protein purification was performed by Nickel chelate affinity chromatography under denaturing conditions, according to the manufacturer's protocol (Qiagen).

### Enzymatic activity assay of deoxyhypusine synthase expressed from *P. vivax*

Deoxyhypusine synthase activity was measured by the incorporation of 40 mM [^14^-C] spermidine into eIF-5A precursor protein. A total volume of 50 μl consisted of 83.3 mM Nickel affinity-purified eIF-5A, NAD^+ ^1 mM, 0.1 M glycine-NaOH buffer, pH 9.5, 1 mM dithiothreitol and Nickel-affinity purified DHS enzyme (1–5 μg). DHS activity assay was performed after a second buffer exchange step on a NAP TM5 column (Amersham Pharmacia). Assays were incubated for 2 h at 37°C. The reaction was terminated by adding 10 ml of 1 M potassium phosphate, pH 6.3, with 60 mM spermidine. The incubation mixture was absorbed onto a Whatman No.3 MM paper disc, and developed using the method of Sasaki *et al*. (1996) [16].

### Western blot analysis of DHS from *P. vivax*

Western blot analysis was performed using the sandwich method in a semi-dry transfer electroblotting apparatus (Fisher Scientific) for 1 h at 25 V. After 3 h of induction, protein extracts were transferred to a nitrocellulose membrane in transfer buffer (12 mM Tris, 96 mM glycine and 20% methanol). The primary polyclonal antibody against human DHS protein was kindly provided by MH. Park (Oral and Pharyngeal Cancer Branch, National Institute of Health, Bethesda). The polyclonal human anti-DHS antibody was diluted (1: 800) in TBS (10 mM Tris, 15 mM NaCl) buffer, incubated for 1 h at room temperature, and washed twice with TBS-Tween buffer. The primary antibody was detected with a secondary immunopure rabbit anti-mouse- IgG- alkaline phosphatase conjugate (Pierce) at a dilution of 1: 10.000. The membrane was washed with TBS-Tween buffer, and detected with nitroblue tetrazolium phosphate (5% in 70% dimethylformamide) and 5-bromo-4-chloro-3-indolylphosphate (5% in 100% dimethylformamide) in alkaline phosphatase buffer (100 mM Tris-Cl, 100 mM NaCl, 5 mM MgCl_2_).

## Authors' contributions

JTN was responsible for cloning and, in part, expression experiments on the *dhs *gene. MN performed experiments on specific enzymatic activity and inhibition. AEK supervised the work, organized financial support, and prepared the manuscript. AH critically appraised the manuscript.

## Figures and Tables

**Table 1:** Determination of DHS activity from *P. vivax *by eIF-5A modification

incubation temp. 37°C	incubation temp. 37°C	incubation temp. 37°C
eIF5A lys	Water	50 μM GC7
^14^[-C]-spermidine	^14^[-C]-spermidine	^14^[-C]-spermidine
1268 U DHS/mg protein	n.d. U DHS/mg protein	35 U DHS/mg protein
*P. vivax *DHS	*P. vivax *DHS	*P. vivax *DHS
**A**	**B**	**C**

column A) complete enzymatic assay B) control: DHS enzyme was substituted by water C) inhibition of DHS by the inhibitor GC7 (50 μM).
